# Medical Waste Management in Community Health Centers

**Published:** 2018-02

**Authors:** Jafar Sadegh TABRIZI, Ramin REZAPOUR, Mohammad SAADATI, Samira SEIFI, Behnam AMINI, Farahnaz VARMAZYAR

**Affiliations:** 1. Tabriz Health Service Management Research Center, Dept. of Health Service Management, School of Health Management and Medical Informatics, Tabriz University of Medical Sciences, Tabriz, Iran; 2. Iranian Center of Excellence in Health Management, Dept. of Health Service Management, School of Health Management and Medical Informatics, Tabriz University of Medical Sciences, Tabriz, Iran; 3. Road Traffic Injury Research Center, Tabriz University of Medical Sciences, Tabriz, Iran; 4. Dept. of Health Service Management, School of Health Management and Medical Informatics, Tabriz University of Medical Sciences, Tabriz, Iran

**Keywords:** Medical wastes, Community health center, Wastes management, Primary health care

## Abstract

**Background:**

Non-standard management of medical waste leads to irreparable side effects. This issue is of double importance in health care centers in a city which are the most extensive system for providing Primary Health Care (PHC) across Iran cities. This study investigated the medical waste management standards observation in Tabriz community health care centers, northwestern Iran.

**Methods:**

In this triangulated cross-sectional study (qualitative-quantitative), data collecting tool was a valid checklist of waste management process developed based on Iranian medical waste management standards. The data were collected in 2015 through process observation and interviews with the health center’s staff.

**Results:**

The average rate of waste management standards observance in Tabriz community health centers, Tabriz, Iran was 29.8%. This case was 22.8% in dimension of management and training, 27.3% in separating and collecting, 31.2% in transport and temporary storage, and 42.9% in sterilization and disposal. Lack of principal separation of wastes, inappropriate collecting and disposal cycle of waste and disregarding safety tips (fertilizer device performance monitoring, microbial cultures and so on) were among the observed defects in health care centers supported by quantitative data.

**Conclusion:**

Medical waste management was not in a desirable situation in Tabriz community health centers. The expansion of community health centers in different regions and non-observance of standards could predispose to incidence the risks resulted from medical wastes. So it is necessary to adopt appropriate policies to promote waste management situation.

## Introduction

Health is inalienable right of all people and providing and supplying the people with desired degree of health is a supreme social purpose worldwide. The most important historical events done in the evolution of supply and production of health services is the international community's decision based on admission of Primary Health Care (PHC) to achieve this goal (1). In Alma-Ata Declaration of the 30^th^ World Health Assembly in 1977, the main social purpose of the states up 2000, must be achieving all people to a level of health (physical, mental, and social health) which allows them enjoying an effective and productive life. The method of achieving health for all, PHC, was introduced in 1978 (1–3).

Hence, the governments decided to provide required facilities and possibilities for primary healthcare services. This issue led to an extensive increase in health care centers and hospitals across all cities. However, on the other hand, this had caused to accelerate the process of emerging medical wastes producing centers including hospitals, laboratories, health care centers, clinics, dental offices, and other centers providing medical services as well as increase the amount and variety of medical wastes (4, 5). The wastes of healthcare system are one of the most important environmental problems which was of particular sensitivity due to presence of dangerous, toxic and pathogenic agents such as pathological radioactive, pharmaceutical, chemical, infectious, therapeutic appliances and equipment (6–8).

According to Iranian standards, medical wastes are divided into two categories of normal (quasi-home) wastes, and the wastes resulted from special medical care (9). Medical wastes are regarded as a sub-group of hospital wastes resulted from health services and had the potential risk of infection (10). According to WHO, 10%–20% of medical wastes includes in hazardous and infectious group (11). In Iran's medical waste management regulations, medical wastes are divided into 4 categories: infectious wastes, sharp wastes, chemical and pharmaceutical wastes and normal wastes. Following steps of separating, packaging, collecting, storing, transporting and sterilization of wastes had been regarded as waste management cycle (9).

There are decisive witnesses indicating that some hazardous agents such as HIV, Hepatitis B and Hepatitis C, are transferrable through medical wastes. Therefore, community health centers wastes may play a key role in the pathogenesis, transmission of infection, and environmental contamination (6).

Health services providers and the staff that was in contact with medical wastes had the highest rate of injury while working. Considering, 150–200 out of 1000 person who had a role in medical wastes collecting, had injury experience (12). Therefore, the proper waste management in community health centers may prevent the possibility of out-breaking and spread the diseases and regional epidemics (10, 13). According to the Waste Management Act (WMA) approved in 2004, the producer center is in charge of own wastes sterilization. Since the small health care centers such as offices, vaccination centers, and clinics are not able to sterilize their wastes, due to lack of required equipment (14, 15). The desirable situation of hospitals was indicated in terms of separating, collecting, and transporting and temporary storage section (16).

Most of the community health centers in Tabriz include medical, midwifery, dentistry and vaccination units. This lead to continuous production of medical wastes that lack of proper waste management process could result in irreparable risks to the community. Most of literature on waste management had insisted on medical wastes of hospitals, but wastes management in community health centers had not been seriously considered.

This study aimed to investigate the waste management in Tabriz community health centers.

## Methods

This was a triangulated cross-sectional study (quantitative-qualitative). The study population consisted of all community health centers in Tabriz (census), northwestern Iran including 58 centers. Thirty-three health centers contained general practitioner, midwifery and vaccination units and 25 centers contain a dentistry unit, additionally. Data collection was done in winter 2015.

Ethical Committee of Tabriz University of Medical Sciences approved the study.

The quantitative part was performed using cross-sectional design. Data collection was done through a researcher made checklist based on Iranian medical waste management standards. The checklist was assessed by 10 experts [(including 2 Environmental Health experts (experts in the field of waste management), 2 experienced managers in community health centers and 2 experts in the field of Health Services Management)] for validity (CVI=0.82).

The checklist included 4 dimensions of “Management and training”, “Sterilization and disposal”, “Collecting and separating” and “Transporting and temporary storage” in medical waste management process.

The checklist was completed by the researchers through a site visit and document reviewing in health centers. Moreover, in some cases, interview was done by the person who was in charge of waste management in health centers. Questions were scored by assigning a score of one to standards compliance and zero to non-compliance. Data were analyzed using SPSS (ver.19, Chicago, IL, USA).

The qualitative part was carried out by researcher observation method. All the observations were recorded by researchers through the data collection process in community health centers. Content analysis was used to analyze the recorded observations. Moreover, where it was possible, the quantitative data was supported by the qualitative data.

## Results

**Fig. 1:**
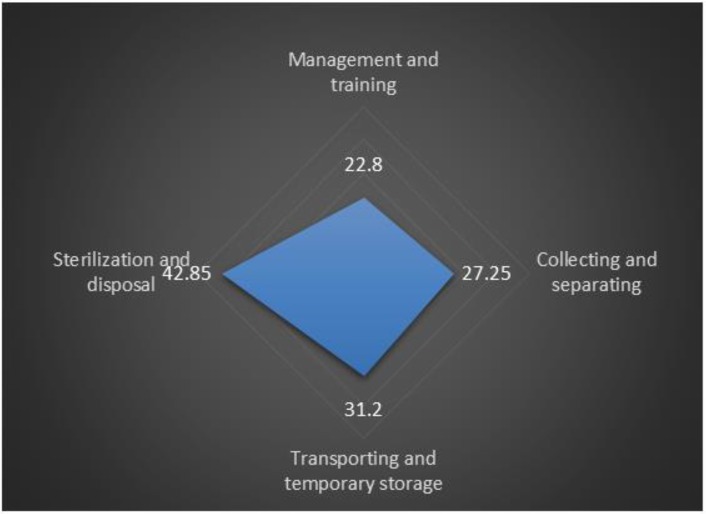
Standard observance rate in medical waste management dimensions

The data were collected from 57 community health centers in Tabriz. There were no recorded documents about the wastes quantity. Although, the wastes were contributed of sharp wastes and infectious wastes from Medical, Dentistry, Laboratory and Midwifery activities. Generally, the average of waste management standards observance in Tabriz community health centers was calculated as 29.82±8.24. The following results were obtained regarding the situation of waste management in four dimensions of waste management process (Fig. 1).

### Management and training

Actually, only 3.5% of health centers had a standard observance average upper than 50% in this dimension. Moreover, 59.6% had rate lower than 25%.

About 98% of the health centers had no plan for waste management. Empowerment workshops on waste management knowledge were held for staff only in 10.5% of centers at least once a year.

### Collecting and separating:

**Fig. 2:**
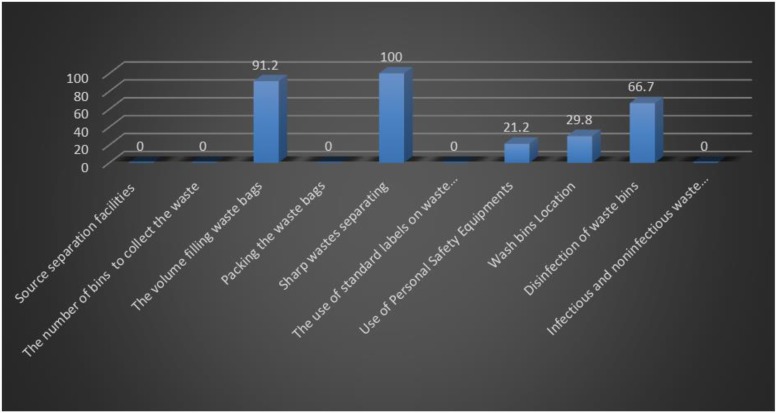
The standard observance rate for each item in wastes collecting and separating dimension

Nearly 29.8% of the health centers, had a standard observance rate lower than 25%. Moreover, 70.2% of the health centers, had the rate of 25%–50%, and standard observation rate was upper than 50% in none of the health centers. Fig. 2 shows the observation of each item in this dimension in health centers.

In all centers, different types of bins and bags with different colors had been used for general wastes, but special containers (safety boxes) had used to separate sharp and cutter wastes. Moreover, general and infectious wastes were thrown in common waste bins and were not separated properly. Almost in all centers with laboratory, its wastes including urine, feces and blood samples, were not disposed of properly.

### Transporting and temporary storage

In most of the health centers (43.9%) standards, observance was less than 25%. only in 17.5% of centers, standards observance rate was above 50%.

No special place was allocated to temporary storage of infectious wastes and safety boxes in 61.4% of centers. Moreover, the primary storage place of the medical wastes had not desirable situation in any of centers. Staircases, under the stairs, one of the center rooms (like vaccine room, reception or midwifery room), lavatory and even in some cases balcony of centers were used as the place of temporary storage in community health centers. Mean of wastes storage time, in temporary storage place, was obtained about 1436.245± 1165.06 h, 24 times more than standard.

### Sterilization and disposal

Health centers had no documents for recording the volume of sterilized wastes per each working time of the sterilization device, delivering them to municipality, or the volume of accumulated safety boxes in the centers. Sterilized wastes did not transport by municipality workers, routinely. Autoclave was used to disposal infectious waste whereas microbial tests did not perform to assess the performance of autoclave. Tabriz health centers had a standard observation rate of 42.85% in medical wastes sterilization and disposal.

## Discussion

The situation of waste management in Tabriz community health centers was not at an acceptable level (less than 50%) in all four dimensions of medical wastes management process. Due to high dangerous nature of medical wastes produced in health centers, these wastes should be considered seriously, and the required measures are done to separate and isolate them. Rate of standard observation in “Management and Training” and “collecting and separating” dimensions was 22.8% and 27.3%, respectively. This issue suggests very little attention to wastes proper disposal in health centers which are in charge of promoting people health. Waste management training is a necessity for health care managers and employees (17). The standard observation rate in mentioned dimensions in Tabriz public hospitals were 59.62% and 47.38% and in private hospitals were 71.88% and 56.25%, respectively. This indicates the hospitals pay more attention to waste management process (18). This may be due to the accreditation standards implemented in hospitals.

“Management and training” dimension is the main and influencing element in waste management process and its better function could improve the whole process performance. There was no specific and comprehensive waste management plan in community health centers. The management and training dimension was at a week level in Bandar Abbas health centers (19).

The existence of public wastes in the bins allocated for infectious wastes and lack of proper separation of medical wastes had led to entering dangerous wastes to the community waste disposal cycle. This may result in irreparable environmental dangers due to the lack of sterilization. Regarding, separating the infectious wastes and public wastes is completely necessary. However, the results indicated too weak circumstances of collecting and separating medical wastes standards observation. Moreover, the required infrastructures, tools and equipment for medical wastes separating were not provided adequately in Tabriz health centers. The state of separation was “very undesirable” and only collecting of syringe and sharp objects had desirable situation in Tehran hospitals (20).

However, storage and sterilization of medical wastes should be done properly, otherwise, all measures and endeavors on wastes separation would be null and invalid. Standards observation rate in dimensions of “transport and temporary storage” and “sterilization and disposal” were 31.2% and 41.8%, respectively. Standards observation rate of 47.2% and 80% in mentioned dimensions were reported in public hospitals, and 56.3% and 85% in private hospitals, respectively (18). Moreover, collection, separation and transportation of medical wastes in Tehran public hospitals were in an acceptable status than disposal (21). Due to the extremely high importance of waste management in hospitals accreditation standards, required structures have been provided in hospitals. But such cohesion had not observed in primary health care system.

Sterilization and disposal of sharp wastes are one of the most important steps in medical wastes management. Despite that, this issue was seriously considered by community health centers and observed standards were relatively high, it was not at an acceptable level. Furthermore, sterilization and disposal of medical wastes in Tehran health centers were “undesirable” (20). Similar results were reported in Tehran public hospitals (21).

## Conclusion

Waste management status was unfavorable in Tabriz community health centers. Standards observation in “Management and training” and “collecting and separation” dimensions were worse than other dimensions. Besides the waste management educations, proper policy procedures must be taken into action in order to improve the waste management process. Moreover, employing a trained staff as a responsible for waste management process would lead to more safety in the process. Finally, study results call for immediate attention for medical wastes management in PHC system neglected.

## Ethical considerations

Ethical issues (Including plagiarism, informed consent, misconduct, data fabrication and/or falsification, double publication and/or submission, redundancy, etc.) have been completely observed by the authors.

## References

[1] WHO (1978). Declaration of Alma-Ata: International Conference on Primary Health Care, Alma-Ata, USSR, 6–12 September 1978. Retrieved February, 14: 2006.

[2] Khayati F Saberi MH (2009). Primary Health Care (PHC) an ever strategy for health equity extension. Journal of Health Administration, 12:33–40.

[3] WHO (2000). The world health report 2000: health systems: improving performance. ed. World Health Organization.

[4] Damghani AM Savarypour G Zand E Deihimfard R (2008). Municipal solid waste management in Tehran: Current practices, opportunities and challenges. Waste Manag, 28:929–934. 17881212 10.1016/j.wasman.2007.06.010

[5] Sarkar S Haque MA Khan TA (2006). Hospital waste management in Sylhet city. ARPN Journal of Engineering and Applied Sciences, 1:32–40.

[6] Chaerul M Tanaka M Shekdar AV (2008). A system dynamics approach for hospital waste management. Waste Manag, 28:442–449. 17368013 10.1016/j.wasman.2007.01.007

[7] Dehghani M OMRANI GA Nadafi K Marosi M Azam K (2011). Solid waste management in physicians’offices in sabzevar. Hakim, 14:57–63.

[8] da Rocha GC Serra-Freire NM (2009). Paleoparasitology at “Place d’Armes”, Namur, Belgium: a biostatistics analysis of trichurid eggs between the Old and New World. Rev Bras Parasitol Vet, 18:70–4. 19772780 10.4322/rbpv.01803013

[9] Commission of infrastructure iate (2008/03/09) Waste Management Regulations. In: education MoHaMe. 1901/56061, Tehran

[10] Prüss A Giroult E Rushbrook P (2014). Safe management of wastes from health-care activities. ed. World Health Organization, Switzerland.

[11] Mohamed LF Ebrahim SA Al-Thukair AA (2009). Hazardous healthcare waste management in the Kingdom of Bahrain. Waste Manag, 29:2404–2409. 19380221 10.1016/j.wasman.2009.02.015

[12] Abedi T Vaezzade F (2002). Hospital wastes management. Rasht: Gap:35–49.

[13] Ngwuluka N Ochekpe N Odumosu P John SA (2009). Waste management in healthcare establishments within Jos Metropolis, Nigeria. Afr J Environ Sci Technol, 3:459–465.

[14] Ashrafi D (2006). Evaluation of medical waste management in Rasht City [dissertation]. Tehran: Tehran University Medical Sciences.

[15] Dehghani M Azam K Changani F Fard ED (2008). Assessment of medical waste management in educational hospitals of Tehran university medical sciences. J Environ Health Sci Engin, 5:131–136.

[16] Dehghani M Fazelinia F Omrani GA Nabizadeh R Azam K (2011). Investigation of Management Status on MedicalWastes in Public Hospitals of Arak City. Ira J Health Environ, 4:93–104.

[17] Ozder A Teker B Eker HH Altındis S Kocaakman M Karabay O (2013). Medical waste management training for healthcare managers-a necessity? J Environ Health Sci Eng, 11:20. 24499642 10.1186/2052-336X-11-20 PMC3776293

[18] Jafar T Hassan T Behnam A Ramin R Samira S Farahnaz V Hasan A (2013) Analysis of the status of waste management in the city of Tabriz hospitals. Thesis. Tabriz University of Medical Sciences. Health Services Management Department.

[19] Koolivand A Mahvi A Azizi K Binavapour M Alipour V (2010). Quality analysis and management of health-care Waste-Products. Bimonthly Journal of Hormozgan University of Medical Sciences, 14:72–79.

[20] Jouhari Z Ramezan K Zaeri F (2008). Waste Management In Health Care Center In Tehran City. Daneshvar Medicine, 15:9–14.

[21] Malekahmadi F Yunesian M (2014). Analysis of the healthcare waste management status in Tehran hospitals. J Environ Health Sci Eng, 12:116. 25426295 10.1186/s40201-014-0116-4 PMC4243780

